# Conservation of Archaeological Bones: Assessment of Innovative Phosphate Consolidants in Comparison with Paraloid B72

**DOI:** 10.3390/nano12183163

**Published:** 2022-09-13

**Authors:** Andrea Díaz-Cortés, Gabriela Graziani, Marco Boi, Lucia López-Polín, Enrico Sassoni

**Affiliations:** 1 Catalan Institute of Human Paleoecology and Social Evolution (IPHES), Zona Educacional 4, Campus Sescelades URV (Edifici W3), 43007 Tarragona, Spain; 2 Departament d’Història i Història de l’Art, Universitat Rovira i Virgili, Avinguda de Catalunya 35, 43002 Tarragona, Spain; 3 Biomedical Science and Technology and NanoBiotechnology Lab, IRCSS Istituto Ortopedico Rizzoli, Via di Barbiano 1/10, 40136 Bologna, Italy; 4 McDonald Institute for Archaeological Research, University of Cambridge, Downing Street, Cambridge CB2 3ER, UK; 5 Department of Civil, Chemical, Environmental and Materials Engineering (DICAM), University of Bologna, Via Terracini 28, 40131 Bologna, Italy

**Keywords:** hydroxyapatite, ammonium phosphate, microhardness, scratch test, scotch tape test

## Abstract

Aqueous solutions of diammonium hydrogen phosphate (DAP) have been recently proposed for consolidation of archeological bones, as an alternative to traditional products. Here, we investigated several routes to improve the performance of the DAP-based treatment, namely increasing the DAP concentration, adding calcium ions and adding ethanol to the DAP solution. Archaeological bones dated to about 1–0.8 million years ago were used for the tests. After preliminary screening by FTIR microscopy and FEG-SEM among different formulations, confirming the formation of new hydroxyapatite phases, the most promising formulation was selected, namely a 3 M DAP solution. The strengthening ability of this formulation was systematically compared to that of the most widely used commercial consolidant, namely Paraloid B72. The performance of the two treatments was evaluated in terms of Knoop and Vickers microhardness, resistance to scratch and resistance to material loss by peeling off. The results of the study show that the DAP treatment was able to improve the bone surface properties and also the resistance to material loss by peeling off, which is more dependent on in-depth consolidation. Paraloid B72 led to the formation of a layer of acrylic resin on the bone surface, which influenced the mechanical tests. Nonetheless, Paraloid B72 was able to penetrate in depth and substantially decrease the material loss by peeling off, even more effectively than DAP. The results of this study indicate that the potential of the DAP treatment for bone consolidation is confirmed.

## 1. Introduction

Archaeological bones are important remains that archaeologists and palaeontologists use to reveal information not only about hominins and their behaviour, but also about evolution of the species, paleo-environment, chronology, and genetic data.

Bone is divided, chemically and hierarchically, into an inorganic component (biological apatite) and an organic matrix (collagen Type I) embedding the apatite crystals [1,2,3]. While buried in the soil, bones experience severe deterioration processes. Soil pH and hydrology, redox values, and the action of microbes can damage one or both the organic and the inorganic fractions, which leads to an increase in porosity and redistribution of the mineral matrix [1,4,5,6]. Due to these modifications, bones lose their internal cohesion and undergo material loss and pulverisation, so that consolidation treatments become essential to guarantee the bone conservation and the possibility to carry out scientific research. In fact, archaeological bones are destined to be continuously handled, beginning with post excavation processes, typically including washing, labelling, documenting, and cataloging. Afterwards, archaeologists and palaeontologists study the remains of bones, which also requires intense manipulation [7,8].

Among bone consolidants, Paraloid B72 (PB72) is the most widely used product. For instance, it has been applied on hominin skeletons, such as the *Australopithecus africanus*, known as “Little foot”, or the lower Pleistocene hominin *Homo antecessor* [7], as well as to faunal remains [9,10]. PB72 is an ethyl-methacrylate copolymer, which is dissolved in a suitable solvent (typically acetone, with a 5 *w*/*v* concentration) and then applied onto the bones by brushing, dripping or immersion. PB72 exhibits several advantages, such as rapid curing and, in principle, re-treatability, since PB72 could be theoretically removed by application of a solvent (e.g., acetone) [11,12,13,14,15]. However, PB72 also has some limitations, such as its incompatibility with a humid environment and wet substrates [16,17] and a glass transition temperature of 40 °C [18], which could be an issue when consolidation treatments need to be applied in situ. Additional drawbacks that have been reported when PB72 is used for consolidation of heritage stones exposed outdoors (e.g., color change, sensitivity to UV radiation, alteration of the transport properties of the substrate, poor outdoor durability) may not be as important in the case of archaeological bones, since they are usually stored indoors in collections, where environmental parameters are controlled. Moreover, even though some studies have shown that radiocarbon dating of bones treated with PB72 is feasible after cleaning the samples [19], another possible limitation of PB72 is that it may interfere with radiocarbon dating of the bone remains, as it is a source of exogenous carbon [20]. In any case, bone remains older than 40–50 thousand years can hardly be dated reliably by this technique [21].

To overcome the limitations exhibited by PB72, in the last two decades research has focused on alternative inorganic consolidants, initially developed for heritage stones, plasters or wall paintings and later tested also for archaeological and palaeontological bones and fossils. Inorganic consolidants tested on bone remains include TEOS-based products [22], nano-dispersions of calcium hydroxide [23] and ammonium phosphate solutions [24,25,26]. In spite of the search for innovative consolidants, PB72 still remains the product most widely used by practitioners for consolidation of archaeological, palaeontological and fossil bones [15,27,28,29,30,31], hence, evaluating the performance of new consolidants against that of PB72 is very important. 

Among the innovative consolidants proposed in the last few years, aqueous solutions of diammonium hydrogen phosphate (DAP, (NH_4_)_2_HPO_4_) show a high potential [24,26,32], especially considering the similarity and, hence, the compatibility between the hardened consolidant and the inorganic component of bone. Indeed, the hardened consolidant is ideally hydroxyapatite (HAP, Ca_10_(PO_4_)_6_(OH)_2_), although deviation from the stoichiometric formula may occur because of the incorporation of foreign ions, such as CO_3_^2-^, which is, indeed, the most frequent ionic substitution in bone. The inorganic component of bone is, in fact, biological apatite, having general formula Ca_9.3_X_0.7_(PO_4_)_4.3_(HPO_4_,CO_3_)_1.7_(OH, CO_3_)_0.3_Y_1.7_, where Y may indicate hydroxyl, fluorine or chlorine ions, while X indicates additional substitution by magnesium, strontium, manganese, silicon or zinc ions [33,34,35]. 

Several formulations of the DAP-treatment have been tested on several types of bone remains. A few studies have investigated the use of DAP solutions alone, considering a range of different concentrations (0.5, 1 and 2 M DAP in [24], 1 M in [26]). To increase the consolidating efficacy and possibly favour formation of HAP, the pre-treatment of the bone remains with a suspension of Ca(OH)_2_ nanoparticles before application of the DAP solution has also been explored [26,32]. As an alternative route [36], or as an additional preliminary step before application of the nano-Ca(OH)_2_ dispersion and the DAP solution [20], impregnation with already-formed HAP nanoparticles has also been investigated. In all cases, the DAP-based treatment has provided very encouraging results, in terms of consolidating efficacy and compatibility with the substrate [20,24,26,32]. Importantly, recent findings have pointed out that the DAP-treatment does not substantially affect the ability to recover endogenous DNA molecules from the treated bones [26] and to perform paleogenetic analysis and radiocarbon dating [20], thus overcoming some of the limitations of Paraloid B72.

In light of the studies briefly summarized above, the aim of the present paper is to extend the literature on the use of DAP-based treatments for conservation of bone remains, by addressing three aspects that have not yet been fully explored in the literature:

- ***Formulation of the DAP treatment***. To increase the strengthening efficacy, studies reported in the literature have focused on pre-treatment with suspensions of nano-Ca(OH)_2_ [26,32] and/or nano-HAP [20]. However, several alternative strategies to improve the consolidating efficacy of the DAP treatment, when applied onto heritage stones and mortars, have been proposed in the literature (e.g., increasing the DAP concentration [37], adding Ca^2+^ ions [38] or adding alcohol to the DAP solution [39,40]), but they have not yet been tested in the case of bone conservation.
- ***Systematic comparison with Paraloid B72***. Even though a comparison between DAP and PB72 has been reported in one of the first studies on the topic [24] and, recently, PB72 has been compared to the DAP-based treatment in terms of impact on radiocarbon dating [20], still, to our best knowledge, no systematic comparison between increases in bone mechanical properties brought about by DAP and PB72 has been reported in the literature.
- ***Choice of the archeological samples.*** The majority of the studies reported in the literature has been carried out on fresh bone and/or bone powders, while studies on archeological bones have been mostly limited to specimens up to 3000 years old. Here, we applied the treatments on archaeological bones dating to 1–0.8 million years ago, which corresponds to a very challenging state of conservation.

Therefore, in the present study we first compared several formulations of the DAP treatment, differing in terms of DAP concentration, addition of CaCl_2_ as a calcium source and addition of ethanol, the effects of which have never been tested for bone conservation. Then, based on the composition of the new consolidating phases and the apparent increase in cohesion, the most promising formulation of the DAP treatment was selected and its ability to increase bone surface hardness, resistance to scratch and resistance to material loss by peeling off were evaluated and systematically compared to Paraloid B72.

## 2. Materials and Methods

### 2.1. Bone Samples

Archaeological bones coming from the Barranc de la Boella site (La Canonja, Tarragona, Spain) were used for the tests. Barranc de la Boella is an open-air archaeological site in a fluvial deltaic context, where water played an important role in the site formation. The bone samples were fragments found in layer 2 of Unit II of Pit I, dated by biostratigraphy and cosmogenic nucleoid to about 1–0.8 million years ago [41]. Based on the size and characteristics of these fragments, the bone samples are believed to be remains of *Mammuthus meridionalis*. Bones from Barranc de la Boella are poorly preserved due to taphonomic alterations (e.g., fissures and powdering), which are a handicap for their archaeological study [42,43] and usually lead to consolidation by Paraloid B72.

### 2.2. Consolidating Treatments

#### 2.2.1. DAP

Diammonium hydrogen phosphate (DAP, (NH_4_)_2_HPO_4_), kindly supplied by CTS s.r.l. (Italy), calcium chloride (CaCl_2_·2H_2_O) and ethanol (EtOH), both purchased from Sigma-Aldrich (Italy, assay > 99%), were used. All water was double deionized.

Based on previous studies on stone consolidation [37], four formulations of the DAP-treatment were initially considered, differing in terms of:

- ***DAP concentration***: previous studies have shown that the higher the DAP concentration, the more abundant the formation of the new consolidating phases and the strengthening efficacy [37], but also the higher the tendency of the new phases to crack during drying [40] and the higher the risk that unreacted DAP remains in the substrate, if not properly removed [44].
- ***Addition of CaCl_2_***. The addition of a calcium source promotes and accelerates formation of the new consolidating phases [38], also having a positive effect on the consolidating ability [45]. Besides formation of HAP, the addition of CaCl_2_ has been found to promote formation of another calcium phosphate mineral, octacalcium phosphate (OCP, Ca_8_H_2_(PO_4_)_6_·5H_2_O) [38].
- ***Addition of alcohol***. The addition of ethanol [39,40] and isopropanol [40] to the DAP solution has been found to be beneficial, as it increases the reactivity of the phosphate ions in solution, thus promoting formation of denser phases (HAP and OCP).

Therefore, the following formulations were considered: (i) 0.1 M DAP + 0.1 mM CaCl_2_ in 10 vol% EtOH; (ii) 1 M DAP + 1 mM CaCl_2_; (iii) 1 M DAP + 1 mM CaCl_2_ in 10 vol% EtOH; (iv) 3 M DAP.

Bone samples were immersed in the various solutions for 24 h (evaporation being prevented by sealing with Parafilm), then extracted and left to dry at room temperature until constant weight. In the case of samples treated with 3 M DAP, following a procedure previously developed for stone [46], the samples were then subjected to a further step, aimed at supplying additional calcium ions to form additional HAP and at removing unreacted DAP that may otherwise remain in the substrate. After immersion for 24 h in the DAP solution and drying, the bone samples were covered with a poultice prepared with cellulose pulp and a saturated solution of Ca(OH)_2_ (1:4 weight ratio), inserting a sheet of Japanese paper between the bone surface and the poultice to prevent residues on the samples. During the first 24 h, the samples were wrapped in a plastic film to prevent evaporation of the Ca(OH)_2_ solution, then the samples were unwrapped and the poultice was left to dry over the samples. Finally, the poultice was removed and the samples rinsed with deionized water. 

Based on preliminary screening tests (cf. Section 3.1), the 3 M DAP formulation was selected as the most promising one and used for the systematic comparison with Paraloid B72, as described in the following. Therefore, in the comparison with PB72, the 3 M DAP solution was labelled as “DAP” (i.e., when no DAP concentration is specified, it is intended to be 3 M DAP).

#### 2.2.2. Paraloid B72

ParaloidB72^®^, kindly supplied by CTS s.r.l. (Italy), and acetone purchased from Sigma-Aldrich (Italy, assay > 99%), were used to prepare a 5% *w*/*v* solution. 

The bone samples were immersed in the solution for 24 h (evaporation being prevented by sealing with Parafilm), then the samples were extracted and left to dry at room temperature until constant weight. This condition was labelled as “PB72”.

### 2.3. Characterization Tests

As described in detail in the following, to identify the most promising formulation of the DAP-based treatment, samples were analysed by FTIR microscopy and FEG-SEM. Then, the consolidating effectiveness of 3 M DAP was compared to that of Paraloid B72, taking untreated bone (labelled “UT”) as reference.

#### 2.3.1. FT-IR Microscopy

The composition of the new phases formed after treatment was analysed by FT-IR, using a Perkin Elmer Spotlight instrument (Italy), coupled to a Spectrum 2 instrument and equipped with a germanium crystal. The following acquisition parameters were used: resolution 4 cm^−1^, 16 scans, scan speed 0.2. Spectra were acquired on 50 × 50 µm^2^ areas.

#### 2.3.2. FEG-SEM Observation

The morphology of untreated and treated samples was observed with a field emission gun scanning electron microscope (FEG-SEM, Tescan Mira3 (Czech Republic), WD = 10 mm, Voltage = 4 kV). Before observation, the samples were made conductive by sputter coating with aluminium. 

#### 2.3.3. Knoop Microhardness

A first indication of the strengthening ability of the two consolidants was obtained by measuring the Knoop microhardness, which has been used in the literature to assess the mechanical properties of a late cretaceous fossil [28]. The test consists in applying a constant load, using a diamond-tipped pyramidal indenter, measuring the size of the print left by the indenter and then calculating the Knoop hardness (HK) according to the formula HK = 14.228 F/D^2^, where F is the load (in kilogram force) and D (in mm) is the long diagonal of the print left by the indenter. 

For each condition, the test was performed by repeating the measurement in 20 spots using a Knoop indenter (Leitz, Italy) with angles of 130° and 172.5°. Taking advantage of the non-destructive nature of the test, the measurements were performed on the same sample before and after consolidation. Two types of tests were carried out:

- to compare the consolidating ability of DAP and PB72, microhardness was determined before and after treatment with the two consolidants (3 replicates per condition), applying a load of 1 N for 30 s.
- to evaluate the effect of rinsing the DAP-treated sample at the end of the treatment and to check that the hardened consolidant was not soluble in water, microhardness was determined before treatment, after the DAP-treatment (before rinsing with water) and then again after rinsing with water, applying a load of 2 N for 30 s.

#### 2.3.4. Vickers Microhardness

To assess the consolidating effectiveness more in depth in the samples, the Vickers microhardness was also determined, as this parameter is often used to assess the strengthening ability of bone consolidants [20,26]. The test was carried out with a Micro Combi Tester (MCT2, Italy) on the same samples used for the Knoop microhardness. In this case, a diamond pyramidal Vickers-type indenter was used in the so-called “trapezoidal loading profile”, applying a load of 1 N and maintaining it for 120 s, with a loading and unloading time of 10 s. The sample hardness, stiffness and elastic modulus were then calculated from the unloading curve using the Oliver-Pahr method [47]. 

#### 2.3.5. Scratch Resistance

Following an example reported in the literature about ivory consolidation [36], the resistance to scratch was also determined [48]. Unidirectional scratching was produced by using a diamond Rockwell C, applied onto the sample surface with a constant load of 0.5 or 1 N with a constant speed of 10 mm/min and a scratch length of 10 mm. For each condition, 4 scratches were made on each sample, two applying a constant load of 0.5 N and two applying a 1N load. The worn tracks were examined to analyse the failure modes of the coated samples. The scratching prints were then observed by SEM (Tescan Mira3) and by a 3D digital microscope (HIROX 8700), which was also used to reconstruct a cross section of the scratch profiles. In the case of the PB72 sample, the digital microscope could not reconstruct the scratch profile, because of the brightness of the sample surface.

#### 2.3.6. Scotch Tape Test

To evaluate the ability of the consolidants to penetrate into the bone samples and provide in-depth consolidation, the resistance to material loss by peeling off was determined through the so-called “scotch tape test” (STT), following a procedure proposed for heritage stones [49]. From a single bone piece, four samples with approximate size 40 × 10 × 10 mm^3^ were sawn. The STT was initially performed on each sample, as reference untreated condition, then 2 samples were consolidated with DAP and 2 with PB72, then the measurement was finally repeated to assess the consolidating efficacy. Each test was carried out by applying a piece of adhesive tape (25 × 8 mm^2^) onto one 40×10 mm^2^ face, taking care of always testing the samples in the same direction, to account for the bone anisotropy. The scotch tape was then manually removed, always adopting the same angle of removal and the same speed. The amount of material removed was then assessed by weighing the sample before and after the test. The STT was repeated 10 times in the same position, to evaluate the permanence of the consolidating efficacy at increasing depth from the surface, as more material was removed after each test.

## 3. Results and Discussion

### 3.1. Identification of the Most Promising DAP Formulation

**Figure 1:**
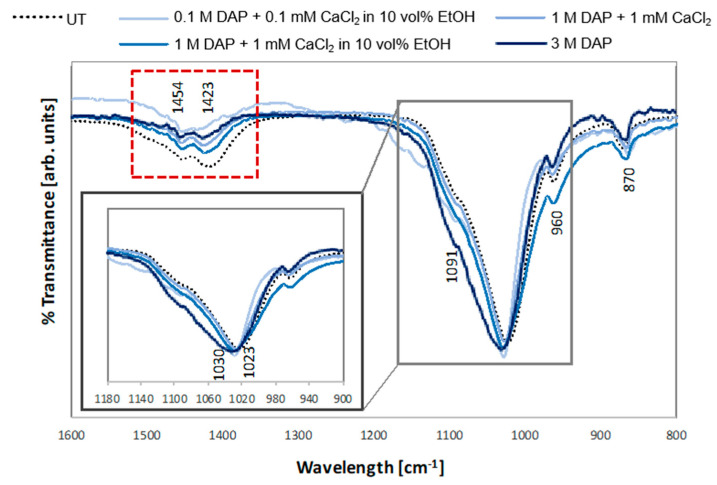
FT-IR spectra of untreated and treated samples.

FT-IR spectra of untreated and treated bone samples are reported in Figure 1. In all the samples, the spectra show bands characteristic of carbonated, calcium deficient hydroxyapatite (CHA), as indicated by the presence of the bands characteristic of phosphates in the 1100–1000 cm^−1^ area, at 1091 cm^−1^ (ν_3_ PO_4_ antisymmetric stretch) and at 960 cm^−1^ (ν_1_ stretching vibration) [50,51], and bands of carbonates at 1454, 1423 cm^−1^ (asymmetrical and symmetrical stretching modes of CO_3_ ν_3_ [52,53]), and at 870 cm^−1^ (ν_2_ CO_3_, characteristic of carbonates and calcium-deficient and/or non-stoichiometric HAP [54]).

Upon treatment, an increase was observed in the area underlying the phosphates stretching band, which indicated formation of additional phosphates phases. The increase was maximum in the case of the 3 M DAP sample. At the same time, a shift was observed in the position of the band from 1020 to 1030 cm^−1^, which indicated the contemporary presence of carbonated hydroxyapatite (deriving from the bone substrate) and newly formed hydroxyapatite, showing lower/no carbonate substitution. Finally, the intensity of the bands of carbonates in the 1400 cm^−1^ area progressively decreased, moving from untreated samples towards treatments at higher concentration, the bands being minimum for the 3 M DAP sample, consistent with a progressive decrease in CHA and increase in HAP. 

All these data together suggested the formation of HAP that was much less substituted than biological apatite as a result of the treatment, especially for the 3 M DAP sample.

**Figure 2:**
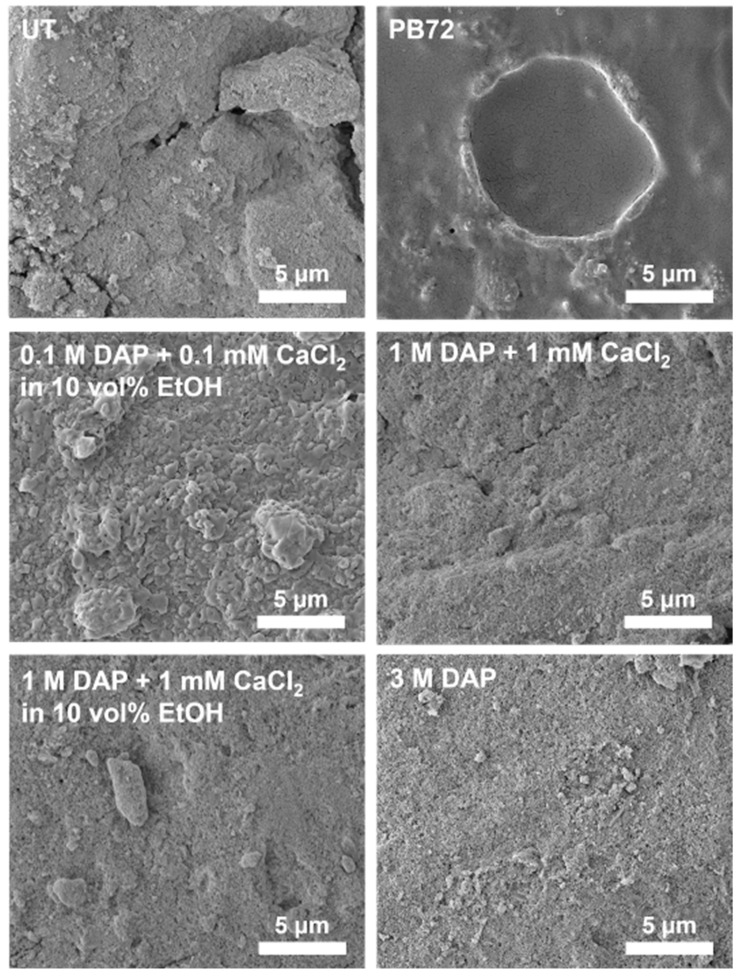
FEG-SEM images of the surface of untreated and treated bones.

A comparison between the surface of untreated and treated samples is shown in Figure 2. 

In the untreated reference (UT), severe fissuring and powdering were clearly visible, consistent with the poor state of conservation of bone remains in the Barranc de la Boella site. After treatment with the four different formulations of the DAP-based treatment, a reduction in pulverization was noticed, although the situation sensibly depended on the analysed area. No clear difference in the morphology of the new phases was visible, but in no case was the bone substrate completely covered with a continuous coating formed of the new consolidating phases. On the contrary, in the case of bone treated with PB72, a continuous crust of acrylic resin was clearly visible over the bone surface, thus masking the underlying bone. In terms of apparent increase in cohesion, the 3 M DAP treatment seemed to provide the best improvement, although the difference with the other DAP formulations was not dramatic. 

Based on FT-IR spectra and SEM images, the 3 M DAP formulation was selected as the most promising one, as it caused an apparent increase in bone cohesion thanks to the formation of new HAP.

### 3.2. Comparison between DAP and PB72

#### 3.2.1. Knoop Microhardness

**Figure 3:**
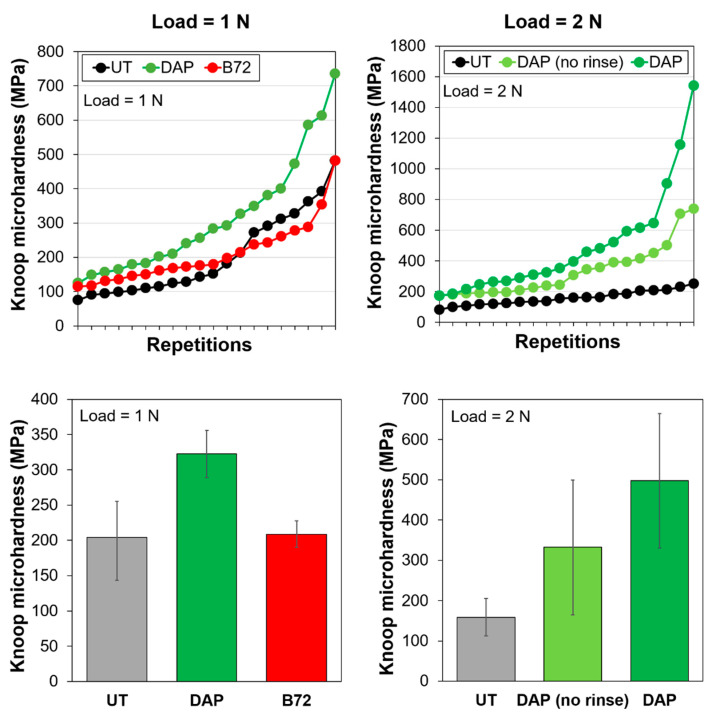
Knoop microhardness of untreated and treated samples. In the case of DAP, microhardness was tested before (pale green) and after (dark green) rinsing with water.

The values of Knoop microhardness of untreated and treated samples are shown in Figure 3.

In the case of the untreated reference (UT), microhardness values exhibited quite high dispersion, in spite of being collected on the same bone fragment, likely as a result of the poor conservation state of the bone samples.

Comparing the performance of the two consolidants (tests with 1 N load), after consolidation with DAP a significant increase in Knoop microhardness was achieved, passing from 204 ± 64 MPa to 322 ± 24 MPa. This increase was due to the formation of the new consolidating phases, which bonded the bone parts more effectively. In the case of PB72, a much lower improvement in Knoop microhardness was achieved (from 204 ± 64 MPa to 209 ± 39 MPa). This could be explained by considering the fact that PB72-treated samples were covered with a layer of acrylic resin (Figure 2). As a result, a modest value of Knoop microhardness was registered, because the indenter had to penetrate through the resin layer, so the microhardness value was highly influenced by the intrinsic low hardness of the resin. A similar effect was also registered in the case of the scratch test (cf. Section 3.2.3).

The test of the durability of the DAP treatment (2 N load) showed that, after rinsing with water, the Knoop microhardness of the DAP-treated sample actually increased (Figure 3), likely because soluble phases, poorly bonded to the substrate (e.g., unreacted DAP), were removed. This confirmed that, at the end of the DAP treatment, rinsing with water is recommended to maximize the treatment outcome.

#### 3.2.2. Vickers Microhardness

**Figure 4:**
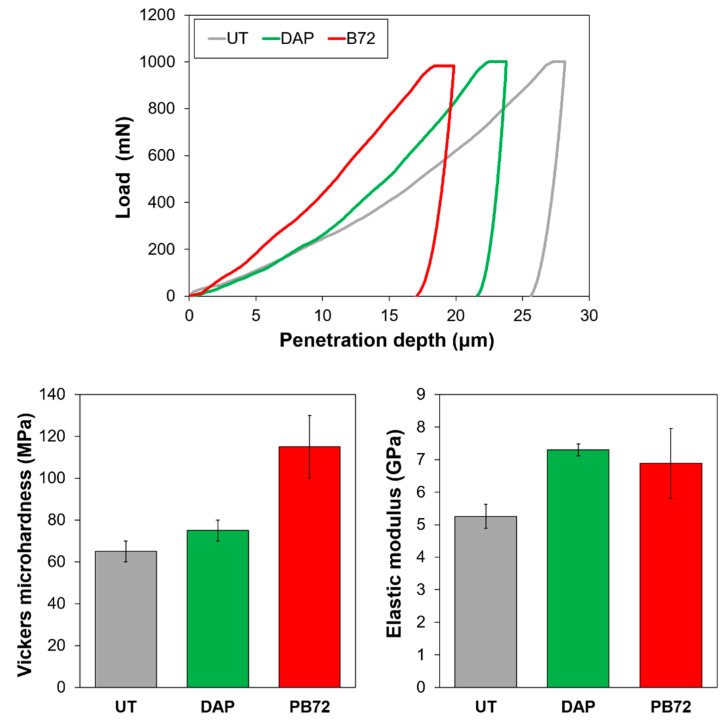
Indentation curves, Vickers microhardness and elastic modulus of untreated and treated samples.

For each treatment condition, a representative loading-unloading curve is illustrated in Figure 4, where the average values of Vickers microhardness and elastic modulus are also reported. 

In the case of the untreated reference (UT), modest values of mechanical properties were registered, in agreement with SEM observations and Knoop microhardness. In particular, the elastic modulus of the UT sample, amounting to about 5 GPa, was sensibly lower than values reported in the literature (e.g., 25–50 GPa in bones from a 10,000 years old site [55]). This was a further indication of the poor preservation state of the bones analysed in this study, as a result of the degradation of the collagen with consequent loss in cohesion and strength [56]. 

Compared to UT, the DAP treatment caused an increase in elastic modulus, although the Vickers microhardness was almost unchanged. On the contrary, PB72 caused an increase in hardness (following a reduction in the depth of the print left by the indenter) and an increase in elastic modulus substantially comparable to that of DAP (Figure 4).

**Figure 5:**
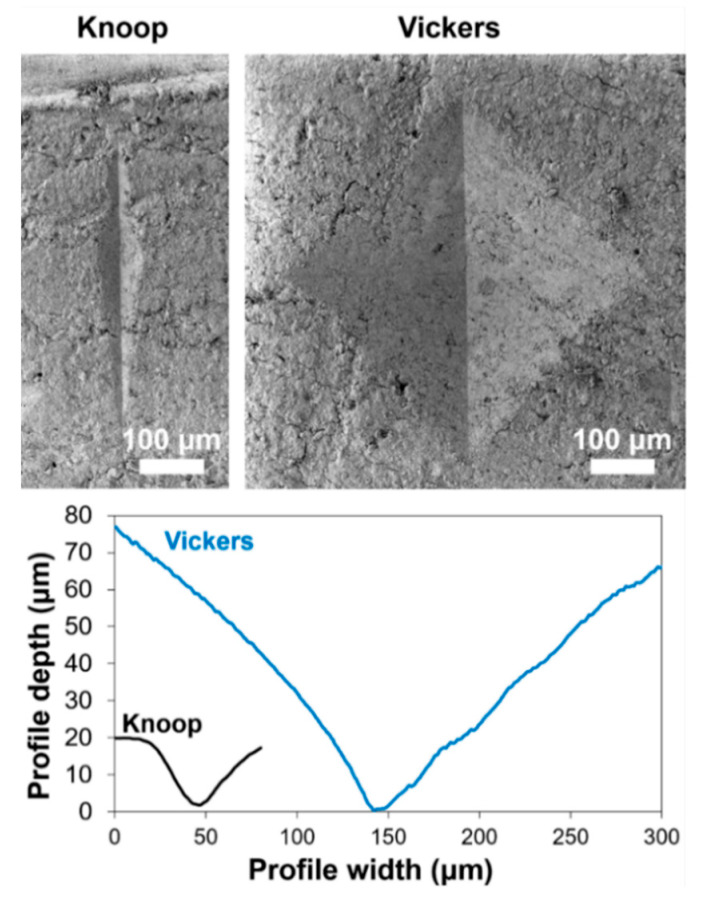
Top views and reconstructed profiles of Vickers and Knoop indenters in untreated bone.

The fact that PB72 produced basically no improvement in Knoop microhardness (Figure 3) but still an increase Vickers microhardness (Figure 4) could be explained by considering the different depth of penetration of the two indenters. As illustrated in Figure 5, the Vickers indenter was able to penetrate significantly more in depth in the sample, compared to the Knoop one. As a result, in the case of bone treated with Paraloid B72, where a surface coating of acrylic resin was present after treatment (Figure 2), the Knoop measurement was essentially limited to the acrylic coating, which led to a scarce surface hardness.

In addition, the difference in the Knoop and Vickers hardness measurements might also be influenced by the properties of the two assays. For Knoop measurements, which exploit a small angle indenter, the mechanical properties mainly reflect the hardness of the measured material, while for Vickers tests, which exploit a large angle indenter, the mechanical properties mainly reflect the deformability of the material. Since PB72 has scarce hardness but relevant elasticity, the results from Knoop and Vickers may differ.

Even though PB72 was able to increase the Vickers microhardness more than DAP, still a slightly lower improvement in elastic modulus than DAP was found (Figure 4). This could be ascribed to the mechanical properties of the two hardened consolidants: while the newly formed HAP had high stiffness (like ceramic materials in general), acrylic resin had low stiffness (like polymeric materials in general), so that a scarce increase in stiffness of the consolidated bones could be expected. 

#### 3.2.3. Scratching Test

**Figure 6:**
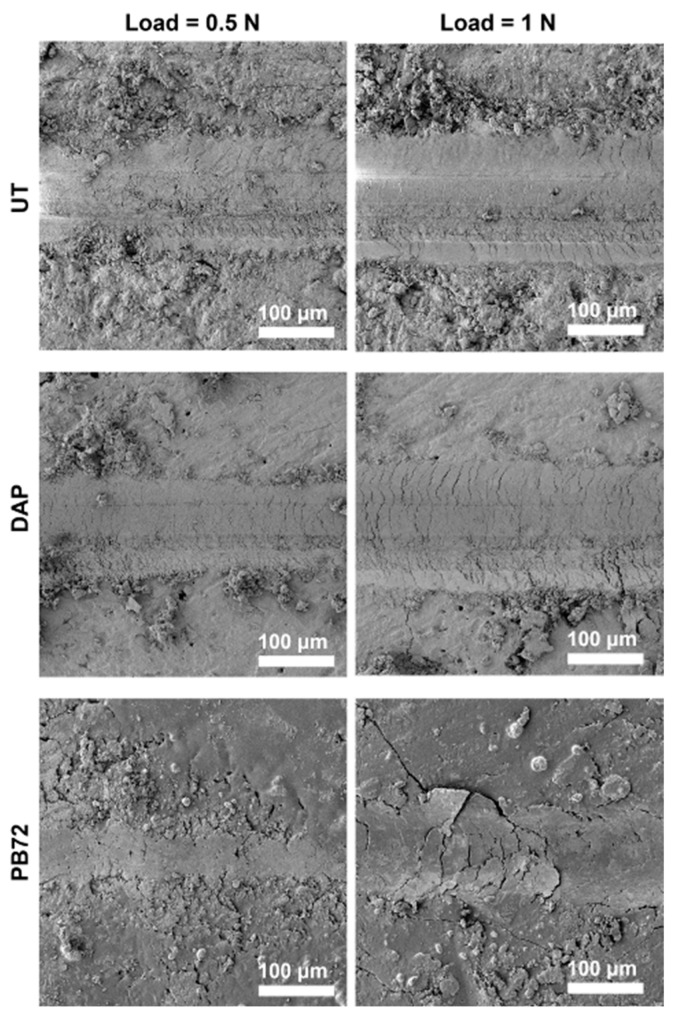
SEM images of scratches on untreated and treated samples.

**Figure 7:**
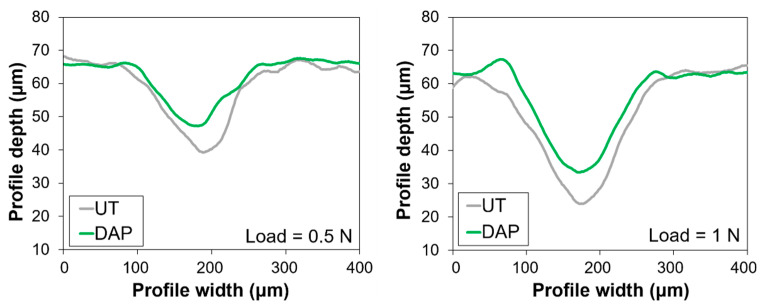
Reconstructed scratch profiles in untreated and treated samples.

SEM images of the scratches produced on untreated and treated samples are illustrated in Figure 6, while a reconstruction of the cross section of the profiles is reported in Figure 7.

In the untreated reference (UT), an increase in the width of the scratch, passing from 0.5 to 1 N, was registered with both microscopes, as expected. After consolidation by DAP, some reduction in the width and in the depth of the scratch was obtained (Figure 6 and Figure 7), although the difference was not dramatic. The DAP samples showed some residues on the edges of the scratch, which might correspond to HAP formed after treatment. In contrast, in the case of the PB72 samples, the scratch was almost imperceptible with load of 0.5 N, while with 1 N load a scratch was visible, with cracks ascribable to the plastic behaviour of the acrylic coating formed over the surface (Figure 2).

The profiles of the scratches reconstructed using the digital microscope (Figure 7) confirmed that the width and the depth of the scratch were slightly reduced after the DAP treatment. In the case of PB72, the measurement could not be performed, as the high brightness of the surface interfered with the measurement.

#### 3.2.4. Scotch Tape Test

**Figure 8:**
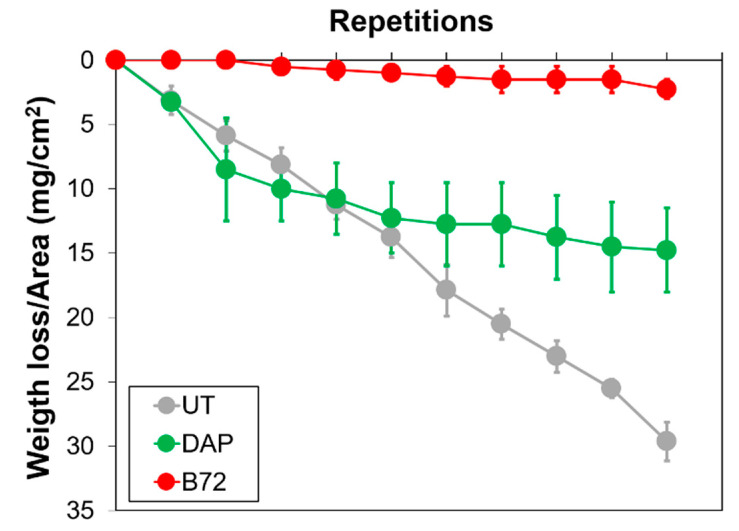
Material loss per unit area after the scotch tape test.

The material loss assessed by scotch tape test, performed before and after consolidation, is reported in Figure 8. Compared to the untreated reference, showing a progressive material loss for increasing repetitions of the test, both consolidants provided substantial strengthening, with some significant difference between the two products. DAP was able to basically halve the material loss (passing from 29.6 ± 1.5 mg/cm^2^ to 14.8 ± 3.2 mg/cm^2^), thus indicating that the consolidating ability was not limited to the surface but was also in depth in the sample. The PB72 showed a remarkable ability to prevent material loss by STT, reducing the amount of material removed from 29.6 ± 1.5 mg/cm^2^ to 2.3 ± 0.8 mg/cm^2^). Notwithstanding the formation of a layer of acrylic resin over the sample surface (Figure 2), the consolidant was still able to penetrate in depth in the bone and significantly strengthen the loose parts. However, it is worth mentioning two aspects that might have influenced the bonding between the tape and the substrate, thus affecting the STT results: (i) the different surface roughness of the specimens (rougher for the UT and DAP conditions, smoother for PB72); (ii) the different levels of adhesion between the scotch tape (having polar functional groups) and the substrate, resulting in lower binding force in the case of PB72 (having hydrophobic functional groups).

## 4. Conclusions

The present study was aimed at comparing several different formulations of an innovative consolidating treatment for archaeological bones, based on the application of a solution of diammonium hydrogen phosphate (DAP) to form hydroxyapatite (HAP) after hardening. The DAP-based treatment was also systematically compared to Paraloid B72, which is nowadays the most widely used product in the practice of bone consolidation. Based on the obtained results, the following conclusions can be derived:

(1) Among the investigated parameters (DAP concentration, addition of a calcium source or addition of ethanol to the DAP solution), the DAP concentration apparently had the highest impact. In fact, application of a 3 M DAP solution allowed the achievement of a good increase in cohesion, thanks to newly formed HAP, as assessed by micro-FTIR and FEG-SEM. Consequently, the 3 M DAP formulation was systematically compared to Paraloid B72.
(2) In terms of effectiveness, both consolidants exhibited significant consolidating ability. In particular, the DAP treatment increased the bone surface properties (microhardness and resistance to scratch) and also the resistance to material loss by peeling off, which is more dependent on in-depth consolidation. The performance of Paraloid B72 was highly influenced by the formation of a layer of acrylic resin on the bone surface: the measurements of microhardness (especially with the Knoop indenter) and resistance to scratch essentially regarded only the surface coating, with limited influence of the bone substrate. Nonetheless, Paraloid B72 was able to substantially decrease the material loss by peeling off (even more effectively than DAP), thus indicating that the consolidant was able to penetrate in depth in the bone samples.

The present study has shown that both consolidants are able to provide significant strengthening of weathered archaeological bones. The DAP-based treatment may have some advantages, such as compatibility with the bone substrate and the lack of interference with radiocarbon dating of the bone remains, unlike Paraloid B72 [20]. Compared to other alternative inorganic treatments proposed in the literature for bone consolidation (e.g., TEOS-based products [22] or nano-dispersions of calcium hydroxide [23]), the DAP treatment has the advantage of a much shorter curing time (hours instead of days). All things considered, the potential of DAP solutions for bone consolidation was confirmed by the present study. Although our tests did not highlight specific weaknesses of the treatment, further studies are in progress to fully characterize the behaviour of the newly developed DAP-based consolidants when applied to bones. More in detail studies are in progress aimed at: (i) assessing the durability of the treatment in different aggressive environments to simulate exposure in unsheltered and semi-sheltered conditions (i.e., exposure to wet/dry conditions, freezing/thawing and thermal cycles); (ii) quantifying the possible interference with post-consolidation analyses (e.g., radiocarbon dating and proteomics); (iii) exploring the feasibility of applying the treatment on site, for consolidation of bones before removal from the soil.

## Data Availability

The data presented in this study are available upon request submitted to the corresponding authors.
